# HMGB1 Carried by Small Extracellular Vesicles Potentially Plays a Role in Promoting Acquired Middle Ear Cholesteatoma

**DOI:** 10.3390/diagnostics13223469

**Published:** 2023-11-17

**Authors:** Michał W. Łuczak, Karolina Dżaman, Łukasz Zaręba, Katarzyna Czerwaty, Jacek Siewiera, Alicja Głuszko, Ewa Olszewska, Jacek Brzost, Ireneusz Kantor, Mirosław J. Szczepański, Nils Ludwig

**Affiliations:** 1 Department of Pathology and Laboratory Medicine, Brown University, Providence, RI 02906, USA; mluczak1978@gmail.com; 2 Department of Biochemistry, Medical University of Warsaw, 02-097 Warsaw, Poland; lukasz.zareba@wum.edu.pl (Ł.Z.); alicja.gluszko@wum.edu.pl (A.G.); 3 Department of Otolaryngology, Centre of Postgraduate Medical Education, 02-097 Warsaw, Poland; kfrydel@poczta.onet.pl (K.D.); katarzynaczerwaty@gmail.com (K.C.); ireneusz.kantor@gmail.com (I.K.); 4 Department of Hyperbaric Medicine, Military Institute of Medicine-National Research Institute, 00-902 Warsaw, Poland; jacek.siewiera@gmail.com; 5 Department of Otolaryngology, Medical University of Bialystok, 15-276 Bialystok, Poland; ewa.olszewska@umb.edu.pl; 6 Department of Otolaryngology, The Children’s Memorial Health Institute, 00-328 Warsaw, Poland; operacjejb@gmail.com; 7 Department of Oral and Maxillofacial Surgery, University Hospital Regensburg, 93053 Regensburg, Germany

**Keywords:** small extracellular vesicles, cholesteatoma, middle ear, exosomes, HMGB1, RAGE, chronic otitis media

## Abstract

Cholesteatoma is a specific medical condition involving the abnormal, non-cancerous growth of skin-like tissue in the middle ear, potentially leading to a collection of debris and even infections. The receptor for advanced glycation (RAGE) and its ligand, high-mobility box 1 (HMGB1), are both known to be overexpressed in cholesteatoma and play a potential role in the pathogenesis of the disease. In this study, we investigated the role of small extracellular vesicles (sEVs) in carrying HMGB1 and inducing disease-promoting effects in cholesteatoma. No significant differences in the concentration of isolated sEVs in the plasma of cholesteatoma patients (*n* = 17) and controls (*n* = 22) were found (*p* > 0.05); however, cholesteatoma-derived sEVs carried significantly higher levels of HMGB1 (*p* < 0.05). In comparison to sEVs isolated from the plasma of controls, cholesteatoma-derived sEVs significantly enhanced keratinocyte proliferation and IL-6 production (*p* < 0.05), potentially by engaging multiple activation pathways including MAPKp44/p42, STAT3, and the NF-κB pathway. Thus, HMGB1(+) sEVs emerge as a novel factor potentially promoting cholesteatoma progression.

## 1. Introduction

Middle ear cholesteatoma is a disease characterized by the expansion of the keratinizing squamous epithelium. Thus, cholesteatoma tissue is composed of proliferating (rapidly growing and multiplying) squamous epithelium known as matrix which is always surrounded by a layer of connective tissue, the perimatrix, infiltrated by inflammatory cells [1]. During disease progression, both innate and acquired responses are important in the middle ear immune system. Damage-associated molecular patterns (DAMPs) are recognized by pattern recognition receptors (PPRs) on the mucosa, inducing an innate immune response. Bellussi et al., suggest that in the inflammatory process of the middle ear, various defects in innate immunity can influence the response, leading to either cure or more severe forms of the disease [2]. HMGB1 (high-mobility group box 1) is a nonhistone nuclear protein, which can act as a DAMP, and is released extracellularly by epithelial and immune cells by stimulating the innate immune system with exogenous molecules, acting as a late mediator of inflammation. HMGB1 is responsible for the cellular stress response triggering T cells, dendritic cells, and endothelial cells, which also secrete HMGB1 in response, ultimately increasing the secretion of pro-inflammatory cytokines. A pathogenic role of HMGB1 was identified in many cancers, chronic inflammatory processes, or even autoimmune disorders [2,3,4,5,6,7,8,9,10,11,12].

HMGB1 is overexpressed in chronic middle ear pathologies and in cholesteatoma, the growth of abnormal tissue can lead to the accumulation of HMGB1 in the affected area [2]. The role of HMGB1 in cholesteatoma is linked to its pro-inflammatory properties. We have previously reported that the expression of HMGB1 protein and its major receptor RAGE (receptor for advanced glycation) is elevated in cholesteatoma samples when compared to normal skin, indicating the potential role of this pathway in specific keratinocyte activation, proliferation, and resistance to apoptosis [12]. Numerous studies have pointed to the role of pro-inflammatory cytokines, such as TNF-α and IL-1 in the pathogenesis of cholesteatoma and bone destruction associated with the disease [13,14]. HMGB1 can stimulate the release of pro-inflammatory cytokines such as TNF-α, IL-1, IL-6, and IL-8 [15]. Especially, IL-1 and TNF-α affect the growth and maturation of osteoclast precursors, leading to subsequent bone resorption [16]. Senda et al., used a model of allergic contact dermatitis to study the role of HMGB1 in keratinocytes and observed an exacerbation of skin inflammation in mice in which the *HMGB1* gene was specifically deleted in keratinocytes, indicating an anti-inflammatory function of nuclear HMGB1 in keratinocytes [11].

Small extracellular vesicles (sEVs) are nanoparticles with diameters of 50–150 nm that are derived from various cells of the organism and are involved in intercellular communication by carrying nucleic acids, proteins, lipids, and cytokines [17]. sEVs are found in various body fluids such as serum, saliva, cerebrospinal fluid, nasal secretion, or semen and can be isolated by several methodologies. In recent years, there has been a very rapid development in research on the role of sEVs in various diseases including inflammatory diseases [18] and cancers [19], but, to date, only a few studies have been published on their importance in the pathogenesis of cholesteatoma [20,21]. Downregulation of miRNA-17 in keratinocyte-derived sEVs of cholesteatoma patients was observed, which may cause increased RANKL levels in fibroblasts and thereby induce osteoclast differentiation, responsible for bone destruction in cholesteatoma [20]. Another study showed that sEVs secreted by human cholesteatoma perimatrix fibroblasts carry low levels of miR-106b-5p, which can promote angiogenesis by binding to the 3’-UTR of the angiopoietin 2 receptor resulting in angiopoietin 2 overexpression [21].

The study was designed to explore cholesteatoma patients’ plasma-derived sEVs concerning their morphological properties, the presence and quantitative assessment of HMGB1, and their functional effects on keratinocyte proliferation and inflammatory cytokine production using keratinocyte cell lines. In our previous studies, we found increased expression of HMGB1 and RAGE in cholesteatoma tissue. Based on these results, we now hypothesize that HMGB1 is also upregulated in sEVs of cholesteatoma patients. We also hypothesize that interaction of cholesteatoma-derived sEVs results in activation of a number of key intracellular signaling pathways that may be involved in cellular proliferation or proinflammatory cytokine production and may play a role in the pathogenesis of cholesteatoma. This is the first study indicating the elevated levels of HMGB1 in sEVs derived from plasma of patients with middle ear cholesteatoma. Despite the existence of an animal model of acquired cholesteatoma [22], there are doubts about its applicability to studying the disease in humans [23]. Due to the inability to create an in vivo model of cholesteatoma, we decided to perform a series of in vitro molecular studies with human keratinocytes to explore the possible role of HMGB1(+) sEVs in the pathogenesis of middle ear cholesteatoma. The results of our studies on the ex vivo model were correlated with the results obtained from in vitro studies, in which we demonstrated the impact of sEVs from cholesteatoma patients on the functions of epithelial cells and the production of the pro-inflammatory cytokine IL-6. Based on our previous and current results from ex vivo and in vitro studies [12,24,25,26], we hypothesize that the increased level of HMGB1 in sEVs in patients with acquired cholesteatoma may be a new and key factor involved in disease progression.

## 2. Materials and Methods

### 2.1. Patients and Samples

Seventeen plasma samples were obtained from seven females and ten males (median age: 42 years; range 31–59 years) with acquired middle ear cholesteatoma who underwent first-time surgery for this reason. The diagnosis of cholesteatoma was confirmed clinically and by histopathological examination. The control group consisted of 7 females and 15 males (median age: 38; range 29–55 years) diagnosed with deviated nasal septum without signs of inflammation and idiopathic sudden sensory neural hearing loss. Peripheral blood samples (10 mL) of patients were collected before any treatment. Plasma was extracted after the centrifugation of peripheral blood at 300× *g* for 10 min, and 2 mL of plasma aliquots were stored at −80 °C until used. The study was approved by the Local Ethics Committees to I.K. (Centre of Postgraduate Medical Education, #15/PB/2018), E.O. (Medical University of Bialystok, #R-I-002/480/2017) and J.S. (Military Institute of Medicine-National Research Institute, #28; 9 June 2019). All participants signed an informed consent, and the study was conducted as recommended in the Declaration of Helsinki.

### 2.2. Cell Lines and Cell Culture

Immortalized human normal keratinocytes cell line (HaCaT) and Normal Adult Human Epidermal Keratinocytes (HEKA) (ATCC^®^ PCS200011™) were acquired from CLS Cell Lines (Eppelheim, Germany) or ATCC (LGC Standards GmbH, Wesel, Germany), respectively. Cells were maintained in RPMI-1640 medium (Sigma-Aldrich, Steinheim am Albuch, Germany) comprising 10% (*v*/*v*) heat-inactivated fetal calf serum (FCS), 2 mM glutamine, 100 μg/mL streptomycin, and 100 U/mL penicillin. Normal Adult Human Primary Epidermal Keratinocytes were maintained in serum-free Dermal Cell Basal Medium (ATCC^®^ PCS200030) supplemented with components of the Keratinocyte Growth Kit (ATCC^®^ PCS200040), which contained the following growth supplements: bovine pituitary extract (BPE), rhTGF-α, L-glutamine, hydrocortisone hemisuccinate, insulin, epinephrine, and apotransferrin. Both cell lines were cultured at 37 °C in an atmosphere of 5% CO_2_. Cells were harvested, washed twice with phosphate-buffered saline (PBS), and detached from culture flasks by brief treatment with 0.02% EDTA solution (Sigma-Aldrich).

### 2.3. Isolation of Small Extracellular Vesicles (sEVs)

To isolate sEVs, 2.5 × 10^6^ cells were seeded with 25 mL of medium in 150 cm^2^ cell culture flasks, as previously described [24,25]. Supernatants were harvested by decanting after 72 h. Preclearing of the supernatants was performed by centrifugation at room temperature (RT) for 10 min at 2000× *g*, followed by centrifugation at 4 °C for 30 min at 10,000× *g*, and subsequent filtration using a 0.22 µm bacterial filter. The filtered supernatants were concentrated at 2000× *g*, and 1 mL of the concentrate was placed on a size exclusion chromatography (SEC) column with Sepharose CL-2B (GE Healthcare Bio-Sciences, Marlborough, MA, USA). The sEVs were eluted in 1 mL fractions using PBS and collected for downstream applications as described elsewhere [25,26]. The SEC columns were prepared for the isolation of sEVs from plasma in accordance with a previously described protocol [26]. Plasma samples were initially purified by centrifugation at 2000× *g* for 10 min at RT, followed by centrifugation at 10,000× *g* for 30 min at 4 °C, and then filtered using a 0.22 μm bacterial filter (EMD Millipore, St. Louis, MO, USA). Subsequently, 1 mL aliquots of precleared serum were placed on SEC columns and eluted with PBS. Fraction No. 4, which is enriched in sEVs, was collected and used for subsequent analysis.

### 2.4. Cryogenic Electron Microscopy (Cryo-EM)

Direct sEV visualization was accomplished using Cryo-EM at the Cryomicroscopy and Electron Diffraction Core Facility (Warsaw, Poland; https://cent.uw.edu.pl/pl/ accessed on 19 September 2023). sEVs were condensed using 100 K Amicon Ultra 2 mL concentrators (Merck, Rahway, NJ, USA) at 4000× *g* for 30 min at RT. A total of 3 µL of concentrated sEVs were plated on lacey carbon EM grids, which were previously glow-discharged (30 s, 25 mA) in a Pelco EasiGlow system, blotted for 2 s, and then plunge-frozen in pre-cooled liquid ethane using Vitrobot (Thermo Fisher, Waltham, MA, USA). The obtained specimens, embedded in a thin layer of amorphous ice, were protected from radiation damage and examined in native state in a 200 kV Glacios Cryo-EM (Thermo Fisher), equipped with a high-sensitive direct electron detector (Falcon 3EC, Thermo Fisher) at accelerating voltage of 200 kV. Images were acquired at a magnification of 72,000× in linear mode with the defocus value in the range of [−2 µm; −5 µm]. The cumulative total dose per image did not exceed 50 e-/A^2^ and a low-dose mode was used to minimize radiation damage during image acquisition. The EPU 2.7 software (Thermo Fisher) was used to analyze single particles.

### 2.5. Nanoparticle Tracking Analysis (NTA)

The evaluation of the size and quantity of sEVs was carried out with a ZetaView, fitted with NTA software (version 2.3, Particle Metrix GmbH, Inning am Ammersee, Germany). Three biological repeats were analyzed for each sample.

### 2.6. Western Blot Analysis

Protein concentrations of the isolated sEVs in Fraction No. 4 were measured using a BCA protein assay (Pierce Biotechnology, Waltham, MA, USA). Proteins were separated by 12% SDS-PAGE under reducing or non-reducing conditions, and 10 µg aliquots/lane of protein were transferred onto a 0.2 μm PVDF membrane (Millipore, St. Louis, MO, USA) and then blocked with 5% fat-free milk. Membranes were incubated with primary antibodies anti-TSG101 (1 μg/mL, Abcam, Cambridge, UK), anti-CD9 (1:1000, Invitrogen, Carlsbad, CA, USA), anti-Grp94 (1:1000, Thermo Fisher), anti-HMGB1 (1:1000, Abcam) and anti-RAGE (1:1000, R&D Systems, Minneapolis, MN, USA) overnight at 4 °C, and then incubated with HRP-conjugated secondary antibody (1:1000 in 5% non-fat milk, anti-rabbit, anti-mouse, Cell Signaling Technology, Danvers, MA, USA) for 1 h at RT. Visualization was performed using ChemiDoc chemiluminescence. For phosphoprotein immunoblotting rabbit polyclonal anti-GAPDH (FL-335) and HRP-conjugated goat anti-rabbit IgG Abs were used (Santa Cruz Biotechnology, Dallas, TX, USA). Western blots were performed using RIPA buffer (Sigma-Aldrich) supplemented with Halt Protease and Phosphatase Inhibitors (Thermo Scientific, Waltham, MA, USA) and PMSF (Sigma-Aldrich) as previously reported [27]. The Western blots were quantified using ImageJ 1.46r software (National Institutes of Health, Bethesda, MD, USA).

### 2.7. Silencing of Toll-like Receptor 4 (TLR4) Expression with Lentivirus Particles

Since HMGB1 is also a ligand for TLR4 in addition to activating RAGE, TLR4 was silenced in HaCaT and HEKA cells using five small hairpin RNA (shRNA) Lentiviral Clones (Sigma-Aldrich) targeting the NM_003266 sequence, as previously described [12]. The TLR4 gene KO was performed following the manufacturer’s protocol [28]. TRC1.5 pLKO.1-puro Empty Vector Control Transduction Particles (Sigma-Aldrich) and TRC1.5 pLKO.1-puro-CMV-TurboGFP Positive Control Transduction Particles (Sigma-Aldrich) were used as missense control and positive control to measure the efficiency of transduction and optimize shRNA delivery, respectively.

### 2.8. Treatment of Cell Lines with sEVs

In all experiments, HaCatT and HEKA cells permanently silenced for TLR4 with lentivirus particles were used. Cells were seeded in wells of 6- or 48-well plates (5 × 10^5^/mL). Subconfluent HaCaT and HEKA monolayers in 48-well plates were starved for 24 h followed by treatment with 3 µg of sEVs per well and incubation for 24 h as previously described [25]. HMGB1 (Sigma-Aldrich) was added at the concentrations of 10–200 ng/mL, and cells were incubated for different periods of time. Cells were also cultured in medium alone or without FCS (controls). In all experiments, HaCaT and HEKA cells permanently silenced for TLR4 with lentivirus particles (Sigma-Aldrich) were used as previously described [12]. For functional assays, plates were incubated at 37 °C for 6 to 96 h. Supernatants were collected and stored frozen at −20 °C for cytokine analyses. Each sample was evaluated in triplicate.

### 2.9. Blocking of High-Mobility Box 1 Effects

In certain experiments, anti-RAGE blocking Abs or isotype control Abs (both from R&D Systems Inc., Minneapolis, MN, USA) were applied to identify whether interference with HMGB1 signaling inhibits cell proliferation or cytokine production as previously described [12]. In preliminary titration experiments, the Ab concentration of 10 μg/mL was found to be able to almost block HMGB1-mediated effects completely.

### 2.10. Cell Proliferation

HaCaT and HEKA cells plated overnight in wells of 6-well or in 96-well plates at the density of 10 × 10^3^ or 2 × 10^3^ per well, respectively, were incubated with fresh medium or medium supplemented with HMGB1, normal control sEVs or cholesteatoma sEVs at various working concentrations. Cell viability and number were determined using microscopy in the presence of trypan blue dye using keratinocytes collected after treatment with TripLE Select solution (Invitrogen) on days 3 and 5 of culture. To confirm sEVs proliferation effects of sEVs on HaCaT and HEKA cells, a colorimetric immunoassay based on the measurement of 5-bromo-12′-deoxyuridine (BrdU) incorporation during DNA synthesis was used according to the manufacturer’s instructions (Roche Diagnostics, Indianapolis, IN, USA) and as previously described [29]. Briefly, after the incubation with sEVs, a BrdU solution (10 µmol/L) was added to each well, and cells were cultured in a cell culture incubator for an additional 2 h. Next, the cell culture medium was removed, fixing solution was added, and cells were incubated for 30 min at RT and then fixing solution was removed. Afterwards, 100 µL of Anti-BrdU-POD working solution was added. After the incubation (70 min at RT), plates were washed three times using the washing buffer. Next, a substrate solution (100 µL) was added, and plates were incubated for approximately 10 min. The reaction was stopped by the addition of 25 µL HCl. The absorbance of the tested samples was read using a spectrophotometer at 450 nm (reference wavelength was 690 nm) (Microplate Spectrophotometer Thermo Scientific).

### 2.11. Measurements of HMGB1 in Plasma or sEVs and Cytokines in Cell Culture Supernatants

Plasma and plasma-derived sEVs lysed with extraction buffer (100 mM Tris, pH 7.4; 150 mM NaCl; 1 mM EGTA; 1 mM EDTA; 1% Triton X-100 and 0.5% Sodium deoxycholate) from cholesteatoma patients and control subjects were used to detect HMGB1 level using an ELISA kit (LifeSpan BioSciences Inc., Lynnwood, WA, USA) in accordance with the manufacturer’s instructions. IL-6 was detected in HaCaT and HEKA culture supernatants using human ELISA kits (BD Bioscences, San Jose, CA, USA) used according to the manufacturer’s instructions as previously described [8,30].

### 2.12. Statistical Analysis

Values are expressed as mean ± standard deviation (SD). Differences between groups were evaluated by Student *t*-test, ANOVA or Kruskal-Wallis one-way analysis of variance. To isolate further differences, appropriate post hoc tests were performed. Differences were considered significant at *p* < 0.05.

## 3. Results

### 3.1. Characterization of sEVs

**Figure 1:**
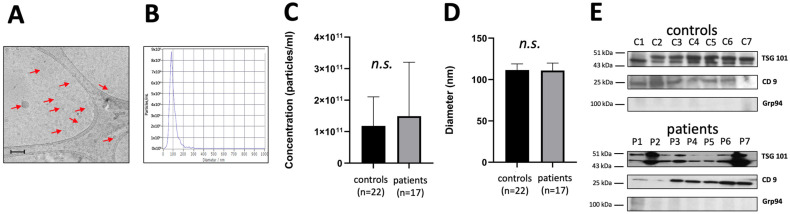
Characterization of small extracellular vesicles (sEVs) from plasma of cholesteatoma patients. (**A**) Representative image of sEVs from Cryo-EM (arrows, bar indicates 200 nm); (**B**) Representative nanoparticle tracking analysis (NTA) plot of the concentration and size distribution of sEVsand particle visualization based on Brownian motion; (**C**) Particle concentration in the cholesteatoma patients and the control group. Results were obtained using NTA; (**D**) Particle diameter in the cholesteatoma patients and the control group. Results were obtained using NTA; (**E**) Representative immunoblotting of the sEV markers TSG101, CD9, and the negative marker Grp94 in sEVs; (no significant difference, n.s.).

sEVs were extracted from plasma or supernatants using SEC and characterized according to MISEV2018 guidelines [17]. Visualized using Cryo-EM, the purified sEVs revealed the typical vesicular morphology with average diameters of 30–120 nm and 4 nm lipid bilayer membranes (Figure 1A). Average particle diameters ranged from 85–120 nm as determined with NTA (Figure 1B). When comparing sEVs isolated from plasma of cholesteatoma patients with sEVs isolated from plasma of controls no significant differences were observed for the concentration of sEVs as well as their diameters (Figure 1C,D). Immunoblotting analysis of sEVs from fraction No. 4 revealed that sEVs carried the sEV markers TSG101 and CD9, together with showing the absence of the negative sEV marker, Grp94 (Figure 1E). 

### 3.2. Levels of HMGB1 in Plasma and in sEVs of Cholesteatoma Patients

**Figure 2:**
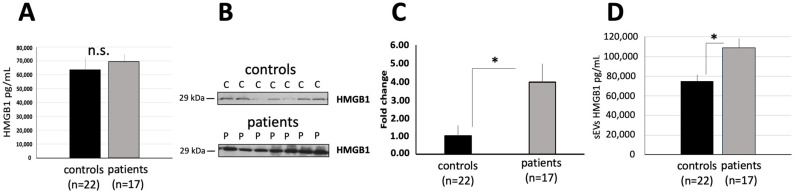
The levels of HMGB1 in plasma and in sEVs of cholesteatoma patients. (**A**) HMGB1 levels in plasma of cholesteatoma patients and controls (no significant difference, n.s.); (**B**) Representative western blots show HMGB1 enriched in plasma-derived sEVs from controls (C1–C7) compared to cholesteatoma patients (P1–P7); (**C**) Semiquantitative evaluation of Western blots using ImageJ 1.46r software; (**D**) HMGB1 levels in plasma-derived sEVs (1 μg) lysed with extraction buffer of cholesteatoma patients and controls, as described in Section 2, (* *p* < 0.05, no significant difference, n.s.).

HMGB1 levels were measured in plasma and in sEVs from cholesteatoma patients and controls. Hereby, we did not find significant differences in plasma levels of HMGB1 when comparing controls and cholesteatoma patients (Figure 2A). As described above, the particle concentration was not significantly different between control and cholestatoma patients, however, western blotting of sEV protein revealed that plasma-derived sEVs isolated from cholesteatoma patients are enriched in HMGB1 in comparison to sEVs isolated from normal controls (Figure 2B). Semi-quantitative analysis of these blots indicated significantly increased levels of HMGB1 in plasma-derived sEVs of cholesteatoma patients relative to the normal control sEVs (fold change: 3.95 ± 1.02 and 1.00 ± 0.58, respectively; *p* < 0.05; Figure 2C). Measuring the levels of HMGB1 in lysed sEVs by ELISA revealed a statistically significant different when comparing cholesteatoma patient-derived sEVs and sEVs isolated from normal controls (*p* < 0.05; Figure 2D).

### 3.3. Characterization of RAGE and TLR4 Expression in HaCaT and HEKA Keratinocytes

**Figure 3:**
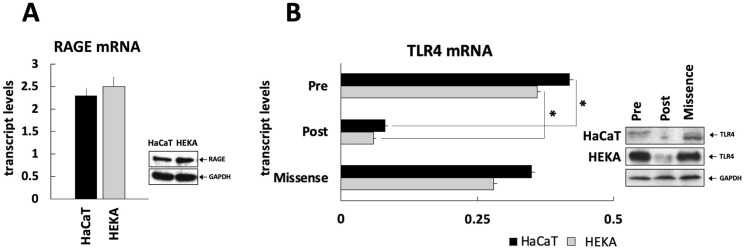
RAGE and TLR4 expression in cultured keratinocytes. (**A**) RAGE expression at the mRNA and protein levels was determined in HaCaT and HEKA cells by RT-PCR and Western blotting; (**B**) Since HMGB1 is also a ligand for TLR4 in addition to activating RAGE, TLR4 was silenced in HaCaT and HEKA cells (as described in Section 2). TLR4 mRNA and protein levels in HaCaT and HEKA cells before and after stable silencing with the lentiviral vector or silencing with scrambled RNA (“missense”), (* *p* < 0.05).

Using qRT-PCR and Western blots, mRNA and protein levels of RAGE were determined in HaCaT and HEKA keratinocytes. Both cell lines expressed RAGE on mRNA and protein levels; however, the expression was more pronounced in HEKA cells (Figure 3A). Additionally, the expression of TLR4 on mRNA and protein level was measured prior to and after stable silencing of the TLR4 gene in HaCaT and HEKA cells. Hereby, TLR4 mRNA and protein expression was reduced by approximately 80% in HaCaT and HEKA cells after gene silencing (Figure 3B).

### 3.4. Plasma-Derived sEVs from Cholesteatoma Patients Promote Proliferation and IL-6 Production in Cultured Keratinocytes

**Figure 4:**
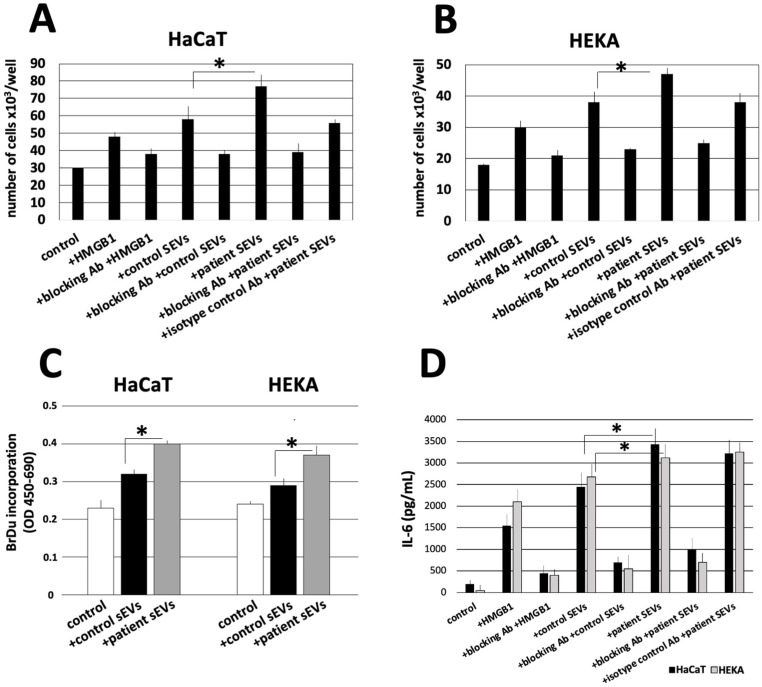
The proliferation and cytokine production of HaCaT and HEKA cells in response to pooled samples of sEVs isolated from the plasma of cholesteatoma patients and controls. HaCaT cells (**A**) and HEKA cells (**B**) were incubated with 3 μg of sEVs at the concentration of 1 μg/μL from the plasma of cholesteatoma patients or from controls. Cell viability and proliferation was assessed after 72 h of culture using microscopy after trypan blue staining. (**C**) BrdU incorporation assay was used (as described in Section 2) in HaCaT and HEKA cells confirming significant differences in the induction of proliferation between normal sEVs and patient sEVs. Data represent mean ± SD from three independent experiments performed in triplicate. (**D**) Levels of IL-6 measured by ELISA assay in HaCaT and HEKA culture supernatants in the presence or absence of HMGB1, RAGE-specific antibodies, and sEVs isolated from the plasma of cholesteatoma patients and controls; IL-6 values were normalized to the 30 × 10^3^ cell count of HaCaT and HEKA cells. All values in this figure represent means ± SD from three experiments with each sample run in triplicate. * *p* < 0.05.

To evaluate the functional effects of plasma-derived sEVs isolated from cholesteatoma patients, co-incubation experiments of sEVs and cultured keratinocytes were performed. In a proliferation assay, the proliferation of keratinocytes was stimulated after treatment with HMGB1, and this effect was partially blocked by using the RAGE-specific antibody in HaCaT and HEKA cells. The co-incubation of keratinocytes with sEVs isolated from the plasma of cholesteatoma patients and controls both resulted in an increased keratinocyte proliferation; however, this increase was significantly higher after treatment with sEVs from cholesteatoma patients (*p* < 0.05). This effect was observed for both keratinocyte cell lines, HaCaT and HEKA (Figure 4A–C). We also observed that sEVs either from normal control or cholesteatoma patients increased keratinocyte proliferation higher than co-incubation with pure HMGB1 protein and that the increase of keratinocyte proliferation by sEVs was efficiently blocked by the blocking antibody indicating a HMGB1-mediated effect (Figure 4A–C).

Besides cell proliferation the levels of the inflammatory cytokine IL-6 were measured in the supernatant of HaCaT and HEKA cells after treatment with sEVs. Hereby, the results were analogous to the proliferation assay, since (1) HMGB1 stimulated the production of IL-6; (2) sEVs isolated from the plasma of controls or cholesteatoma patients stimulated the production of IL-6 even more; (3) cholesteatoma patient-derived sEVs stimulated the production of IL-6 significantly more compared to control-derived sEVs (*p* < 0.05); (4) the induction of IL-6 production was effectively blocked by the RAGE-specific antibody; and (5) the results were consistent for both keratinocyte cell lines (Figure 4C).

### 3.5. sEVs Signaling Engages Multiple Activation Pathways in RAGE-Positive Keratinocytes

**Figure 5:**
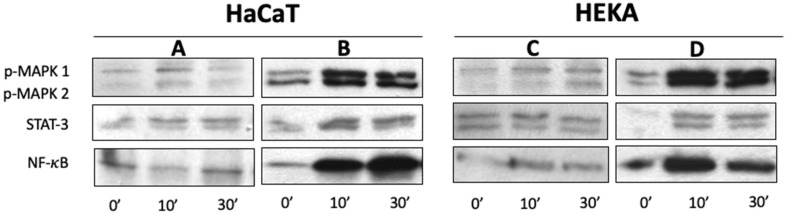
Expression of signaling molecules in TLR4-silenced HaCaT and HEKA cells upon triggering with HMGB1(+)-sEVs as described in Section 2. Western blots of HaCaT or HEKA cells incubated with normal control (**A**,**C**) or cholesteatoma (**B**,**D**) sEVs show dramatic increase of phosphorylation of the MAPKp44/p42, STAT3, and NF-κB after stimulation with cholesteatoma-derived HMGB1(+)-sEVs.

In order to assess the molecular pathways involved in the induction of HaCaT or HEKA cell proliferation and proinflammatory cytokine production upon stimulation with normal control or cholesteatoma sEVs, components of the MAPKp44/p42, STAT3, and the NF-κB pathway components were examined. In our previous study, we have shown that the above mentioned molecules are overexpressed in cholesteatoma tissues [12]. Moreover, all these molecules were constitutively expressed in the nonphosphorylated form in TLR4-silenced HaCaT and HEKA cells [12]. The phosphorylated forms of these proteins were not or only weakly expressed in HaCaT or HEKA cells not stimulated with sEVs. After normal control or cholesteatoma sEV stimulation for 10 to 30 min, phosphoprotein levels weakly (Figure 5A,C) or dramatically increased in HaCaT and HEKA cells (Figure 5B,D), respectively. As this Ab was shown to interfere with HMGB1-mediated induction of proliferation and cytokine production in HaCaT or HEKA cells (see above), our data suggest that HMGB1(+) sEV-induced effects were mediated through phosphorylation of these proteins, and that the effect is much stronger for sEVs isolated from cholesteatoma patients.

## 4. Discussion

The potential efficacy of using HMGB1 as a therapeutic target has been identified in numerous inflammatory diseases. The role of the HMGB1-RAGE axis has been demonstrated in the pathogenesis of chronic rhinosinusitis [6,7,31], as well as in sepsis and sepsis-related organ injury [3], inflammatory skin diseases [9], rheumatic diseases [4], or endometriosis [9]. sEVs carry a variety of factors including proteins, nucleic acids, and lipids and it was demonstrated that these factors are biologically active. In the plasma of patients with malignant diseases, it was shown by large-scale mass spectrometry studies of matching tumor and plasma samples that sEVs carry disease-specific markers and that a large amount of sEVs origins from the primary tumor [19]. This was validated by other studies suggesting that up to 70% of all sEVs circulating in plasma are derived from melanoma cells [32]. When HMGB1 is found in sEVs, it indicates that the protein is transported between cells and HMGB1(+)-sEVs can be released by specific cell types and taken up by recipient cells, where it can exert various effects based on its functional properties. The presence and role of HMGB1(+) sEVs have been studied in various biological processes and diseases, including inflammation, cancer, and tissue repair [26,33,34,35].

HMGB1 carried by platelet-derived sEVs promotes the formation of neutrophil extracellular traps in sepsis and subsequent organ damage [33], and its inhibition in sepsis increases the survival rate [36]. The tumor-promoting effects of HMGB1(+)-sEVs have been confirmed in malignancies, such as gastric cancer [34] and esophageal squamous cell carcinoma [10]. High levels of HMGB1 were found in plasma-derived sEVs of esophageal squamous cell carcinoma patients and were associated with radioresistance of tumor cells [5]. Hypoxia was found to elevate levels of HMGB1 in sEVs isolated from bone marrow-derived mesenchymal stroma cells, ultimately resulting in increased angiogenesis [37]. While most of these studies are based on cell culture-derived sEVs and, therefore, investigate only one population of sEVs in our study, sEVs were isolated from a complex biofluid which contains a variety of sEV subpopulations of different cellular origins. Given the heterogeneity of the sEV samples and also the presence of other factors in the isolated sEVs, HMGB1 might only be one of the sEV-associated factors involved in cholesteatoma pathogenesis. However, incubation of HaCaT cells with HMGB1 has been demonstrated to induce cell proliferation and migration, as well as prevent HaCaT cell apoptosis [12]. It is believed that packaging of HMGB1 into sEVs may protect the protein from degradation and assist in its targeted delivery to specific cell types, thereby enhancing its signaling capabilities [5].

In recent years, the role of HMGB1 in inner ear diseases has also been studied. In the amikacin-poisoned cochlea, temporarily elevated levels of HMGB1 were found in insensitive supporting cells, Deiters cells, as a signal of tissue damage [38]. During the regeneration of spiral ganglion neurons, HMGB1 was re-expressed and translocated into the nuclei of these neurons, potentially promoting repair mechanisms [39]. Using a guinea pig model of noise-induced hearing loss, an increase in HMGB1 expression was observed in the cochlea after noise exposure, while a decrease in HMGB1 expression was noted in the cochlea after pretreatment with dexamethasone [40]. In vitro experiments conducted on a mouse auditory cell line demonstrated that knocking down the *HMGB1* gene could protect cells from damage induced by hydrogen peroxide stress [41]. Moreover, inhibiting HMGB1 using neutralizing anti-HMGB1 antibodies before noise exposure successfully reduced oxidative stress and subsequent inflammation [42]. Additionally, administering an HMGB1-neutralizing antibody directly to the cochlear immediately after noise exposure mitigated hearing loss and outer hair cell death [43].

In this study, we analyzed plasma-derived sEVs isolated from cholesteatoma patients for the presence and quantitative assessment of HMGB1 and to determine the effect of these sEVs on cell proliferation, migration, and inflammatory cytokine production using keratinocyte cell lines. Although an animal model of cholesteatoma using the Mongolian gerbil has been described [22], its applicability to human studies is controversially discussed [23]. Therefore, we used an in vitro HaCaT model to reproduce the cellular and molecular events that might be involved in the development of cholesteatoma. In this study, sEVs isolated from plasma showed normal morphology and their concentrations did not differ between cholesteatoma patients and controls. To date, there have only been two studies in which sEVs have been isolated from cholesteatoma patients [20,21], and our study is the first on plasma-derived sEVs. Hereby, our experiments demonstrated that levels of HMGB1 are elevated in plasma-derived sEVs from patients with cholesteatoma. Analogously, higher expression of HMGB1 was observed in other studies in cholesteatoma tissues or chronic middle-ear pathologies when compared to normal controls [2,12,44]. These correlations may indicate a role for HMGB1 in the formation and development of cholesteatoma.

**Figure 6:**
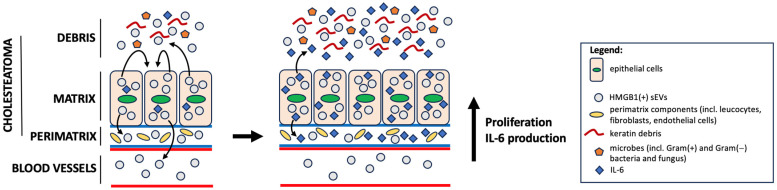
One of the hypothetical models for the progression of acquired cholesteatoma. In the tympanic cavity, there is a proliferation of keratinizing epithelial cells originating from the external auditory canal, which have penetrated the middle ear as a result of chronic perforation of the tympanic membrane (chronic otitis media). This causes local inflammation, uncontrolled development of microorganisms and activation of keratinizing epithelial cells, which, in turn, release enriched HMGB1(+) sEVs into the microenvironment and peripherally. HMGB1(+) sEVs cause, in a paracrine or autocrine mechanism, increased proliferation of keratinizing epithelial cells, as well as the production of the pro-inflammatory cytokine IL-6, which induces a whole cascade of inflammatory mechanisms in the microenvironment of acquired cholesteatoma (e.g., infiltration of pro-inflammatory cells, activation of endothelial cells and fibroblasts, etc.).

We demonstrated using cell lines that sEVs from cholesteatoma patients stimulate cell proliferation of keratinocytes, which is a main mechanism of cholesteatoma pathogenesis [45]. Based on our previous study, it can be concluded that HMGB1 binds to RAGEs, which are overexpressed in keratinocytes, leading to the release of pro-inflammatory cytokines and chronic inflammation [12] (Figure 6).

Moreover, it was demonstrated that the expression of extracellular HMGB1 and DNA fragments in cholesteatoma keratinocytes induce the production of TNF-α and IL-1β, which leads to bone resorption and destruction associated with cholesteatoma [44]. In this study, levels of IL-6 were elevated after stimulation with cholesteatoma-derived sEVs. High levels of IL-6 in patients with cholesteatoma are associated with the injury degree of the ossicle and the poor prognosis of the disease [46,47].

To summarize, research in the field of sEVs and the role of HMGB1 in cholesteatoma is continuously evolving and is considered an area of interest for understanding the disease’s pathophysiology and exploring potential therapeutic targets. In the context of cholesteatoma, HMGB1(+) sEVs can exacerbate the inflammatory condition, potentially leading to tissue damage and complications. A possible limitation of this study is the method for determining the size and counting of sEVs, a concern highlighted in the MISEV guidelines [17]. All methods for isolating sEVs from plasma may also result in the co-isolation of contaminants, such as lipoproteins, in addition to sEVs. Therefore, NTA measurements may count also other non-sEV particles.

## 5. Conclusions

Our data demonstrate that, in cholesteatoma patients, plasma-derived sEVs carry HMGB1 in concentrations which are higher than in sEVs isolated from plasma of normal controls. These sEVs are biologically active and induce functional effects on keratinocytes including stimulation of proliferation and production of pro-inflammatory cytokines. HMGB1(+) sEVs of cholesteatoma patients may also be responsible for inducing systemic inflammatory effects rather than inflammatory responses that are limited to the local temporal bone environment. However, further studies are needed to confirm and extend this aspect of HMGB1(+) sEVs in cholesteatoma.

## Data Availability

Data is contained within the article.
